# From Tissue Engineering to Regenerative Surgery

**DOI:** 10.1016/j.ebiom.2018.01.029

**Published:** 2018-02-19

**Authors:** I. Martin, M. Jakob, D.J. Schaefer

**Affiliations:** Department of Biomedicine and Department of Surgery, University Hospital Basel, University of Basel, Hebelstrasse 20, Basel 4031, Switzerland

The field of plastic and reconstructive surgery has historically been the cradle for the conception and development of tissue fabrication strategies. The compelling need of tissue for challenging reconstructions has also been a fertile soil for the first practical implementation of tissue engineering paradigms (Garfein et al., 2003), which initially occurred in the domains of skin and facial replacements (Cancedda and De Luca, 1993, Puelacher et al., 1994). About 20 years ago, the potential match between clinical expectations in tissue reconstruction and scientific endeavours in tissue engineering has been popularized by the visionary images of engineered cartilage shaped as a human ear under the skin of a mouse (Cao et al., 1997). In the sprouting era of web-based communication, along with enthusiasm towards innovative therapies, that symbol ignited controversial discussions. These were driven by the lay public, critically positioned towards Frankenstein-like creations, and by the scientific community, sceptically considering the underestimated challenges for sound clinical translation. Twenty years later, an interdisciplinary group coordinated by Yilin Cao, the initial promoter of the “ear-mouse”, bursts into the quietened debate and provides evidences that such pioneering concept can lead to a clinical fruition (Zhou et al., 2018).

The study in this issue of *EBioMedicine* describes the use of autologous engineered cartilage grafts for ear reconstruction in one fully documented clinical case and reports treatment of additional four patients with shorter follow-up times. Despite the nature of a preliminary, proof-of-principle study, the work represents an important milestone and offers the opportunity to some general considerations. The authors correctly embed their work in the stream of developments within the major areas required to engineer cartilage grafts, namely cells, signals and scaffolds (Makris et al., 2015). They combined the use of microtia chondrocytes, possibly of enhanced potency due to their neural crest origin (Pelttari et al., 2014), with temporally staggered sequences of growth factors fostering cell growth/differentiation, and with 3D printing/compression moulding technologies to generate scaffolds with adequate shape and stiffness. While these elements represent necessary steps towards clinical translation of the “ear-mouse” vision, it is clear that on their own they would not be sufficient.

The study elegantly describes the surgical tools introduced to manipulate the site of implantation (e.g., tissue expanders, skin grafts) and openly reports the need to adapt the complex surgical procedure to the specific patient conditions, to the extent that three different methods were applied for the five patients treated. In line with similar developments for nasal cartilage restoration (Fulco et al., 2014), the authors confirm the importance to operate a transition from the classical view of tissue engineering, centered on the manufacturing of a suitable graft, towards a more comprehensive paradigm, which can be referred to as “regenerative surgery”. The phrase captures that surgical manipulation of an engineered graft, preparation and conditioning of the environment at the recipient site and reaction of the surrounding soft tissue over time, are at least as critical as the manufactured implant and its designed functionalities.

Emphasis on surgical procedures does not mean neglecting the importance of underlying biological processes. Control of vascularization, management of inflammation, guided tissue regeneration as well as prevention of surrounding soft tissue contraction are some of the concepts exemplifying the critical influence of a surgical procedure on the associated healing events. These need to be more comprehensively understood in order to be more effectively regulated. In this perspective, the bench-to-bed study published in *EBioMedicine* opens several questions which require a bed-to-bench approach. For example, what is the relative contribution of the implanted vs resident cells in the formation of de novo cartilage over time? Could the use of cells be bypassed by delivery of suitable factors recruiting and activating endogenous progenitors? Which signals are required to maintain stability of the formed tissue? Last but not least, the work prompts addressing the fundamental issue of whether the engineered cartilage tissue could grow with the patients, especially since these are young children. Ultimately, in order to guarantee fully coordinated integration, how can an engineered graft be designed to not only replace a body part, but rather activate its development and growth? And towards this goal, should developmental processes be merely recapitulated or somewhat re-engineered, considering that boundary conditions are not the same of an embryo (Tonnarelli et al., 2014)? In this perspective, the terms “tissue engineering”, “regenerative surgery” and “developmental engineering” fuse together and are expected to fuel the translational research in regenerative medicine for the coming 20 years.

## Disclosure

The authors declared no conflicts of interest.
